# Preputial Stenosis

**Published:** 1877-04

**Authors:** James Nevins Hyde


### PREPUTIAL STENOSIS—ITS POSSIBLE COMPLICATIONS AND CONSEQUENCES.

By JAMES NEVINS HYDE, A.M., M.D.

The preputial orifice, when affected with stenosis, relative or absolute, may produce most of the conditions recognized under the names, phimosis and paraphimosis. From complete preputial atresia to slight contraction of the opening, there are various degrees of deformity which correspond, if not in an anatomical, at least in a genetic sense, to the various forms of imperforate, circular and contracted hymen in the female. The prepuce of every male infant is at birth normally in a condition of phimosis; and the question whether or not this condition will eventually result in an abnormal relation of the parts, depends upon the question whether the future development of the glans penis and its investing sac, will be proportionate or disproportionate. According to Van Buren and Keyes,[1] whenever retraction of the prepuce of an infant will permit the glans to be seen, no anxiety need be felt respecting the condition of the parts at puberty.

The recent publication of several papers containing reports of interesting cases, in which phimosis was a prominent element, has directed the attention of the profession anew, to the necessity of correctly estimating the etiological value of this deformity. I have concluded in these pages, therefore, to present a brief digest of some of the interesting features of such cases, without claiming that the instruction which they convey, is either specially new or original.

The direct local results of narrowing of the preputial orifice, differ somewhat according as full retraction is, or is not, possible. In the former case, ablution of the parts is practicable, and thus one source of trouble is removed; still, both conditions may be followed by similar consequences. In adults,

1 Surgical Diseases of the Genito-Urinary Organs, etc., N. Y. 1864 page 13.

where retraction is possible, though slightly painful, the muco-cutaneous opening being more or less tightly stretched as the glans is squeezed through it; the most obvious effects are noticeable upon the ring itself. Here first arise the excoriations, often mistaken for venereal lesions, which may be followed by cicatrization, merely serving to render the ring still less extensible. In this way is often laid the foundation for a severe, obstinately recurring attack of herpes progenialis, or preputialis—a disease which, when fully developed, is as capable of making life utterly miserable as the aggravated forms of eczema ani.

Less obvious is the interference with the development of the penis, which invariably follows. I am quite convinced, from careful observation of many cases, that even the power to fully retract the prepuce does not satisfy the necessities of the organ. For, if the constricting ring be temporarily withdrawn behind the corona glandis, in such individuals, it is speedily followed by a sense of discomfort, and returned to its former position. The glans, then, from childhood to puberty, is compelled to accomplish its development with a membranous hood firmly embracing it—often as firmly as a glove finger, the phalanx. The glans becomes dwarfed, and, as a matter of fact, the size of the body of the penis is proportioned to the size of the glans. Apart from the inconvenience resulting from the irritability of the tender mucous surfaces, thus constantly covered, a grave consequence, and one worthy of consideration, is the mental and moral effect of this deformity upon the individual thus situated. The utterly morbid sensibility of such patients, is only equalled by the condition of hypochondriasis, which accompanies true spermatorrhœa. The following case illustrates this point:

CASE I. E. J., a healthy young man, 22 years of age, five feet eight inches in height, well proportioned, and with finely developed limbs and testes, presented himself for advice in May, 1872, on account of the size of his penis. When flaccid, the organ was but two inches in length, and in circumference no larger than the little finger of a hand of average size. The prepuce could be retracted, but with great difficulty. Upon attempting intercourse, two years before, he had been rallied by his companion upon the diminutiveness of the organ, and since then had been almost constantly brooding over the misfortune, till his mental condition had completely unfitted him for his services as a book-keeper. Circumcision was practiced the following week, and in three months the organ had quite doubled in size.

The best evidence as to the controlling effect of the prepuce in such cases, is the marked and speedy increase in the size of the penis, after the constriction has been, by any method relieved.

When retraction is impossible, the direct consequences are well known to all who have had much experience in the treatment of disorders of the male genital organs. These are, in the infant, "ballooning" of the preputial sac, during micturition, the urine accumulating more rapidly from the opening in the glans than it can escape from the preputial outlet, and collection of sebum which becomes in some cases as hard as the rind of cheese. This collection resembles the hardened vernix in the vagina and cervix of some female children. Subsequently, balanitis, thickening of the mucous membrane, agglutination of the mucous surfaces of the glans and prepuce, suppurative inflammation, vegetations, saline incrustations, preputial calculus, development of parasites, and other complications may arise. The connective tissue of the foreskin soon becomes infiltrated with serum, upon the absorption of which hyperplasia supervenes, and the prepuce becomes inelastic and indurated, or boggy and swollen. In the adult, of course, these same conditions obtain, and are often complicated with chancre, chancroid, gonorrhea, specific balanitis, and lesions of secondary syphilis, such as condylomata, transformed chancres (Fournier), mucous patches, and other syphilides. No small source of trouble in the adult is the consequent soiling of the clothing during micturition. The accumulated urine in the preputial cavity, escapes from its orifice in several contorted streams, whose direction cannot be sufficiently calculated upon to save the clothing, and the consequent inevitable urinous odor which escapes from the person. According to Gross, carcinoma of the penis and prepuce does not result;[1] though Mr. Colles, of Dublin,[2] says upon this point:

1. System of Surgery, Phil. 1872, p. 874.

2. Diagnosis and Treatment of Cancer, London, 1864, p. 251.

"No warty growth upon the prepuce should be neglected in any man of middle age."

It is, however, to the indirect consequences of preputial stenosis that I desire to call especial attention, as the induced phenomena are fully as interesting to the physician as the surgeon. These secondary effects are the result of what is now well recognized as "reflex irritation," and, although authors are not wanting to dispute the sequence of events thus named, still the testimony opposed to their reasoning is almost overwhelming.

The following is a condensed statement of a report made to the Thirtieth Annual Convention of the Ohio State Medical Society, by C. E. Beardsley, M. D., of Ottawa, entitled "Phymosal Paraplegia:"

CASE II. A tall lad, 14 years of age, with dark hair and eyes, extremely emaciated, had loss of power in the lower extremities, inability to hold the head or vertebral column erect, and also suffered from epileptiform convulsions. The latter could be occasioned by pronouncing the word spasm or fit in his presence, or by concussion of the hands, and this as often as he could be aroused from the stupor which followed each attack. He had been thrown from a horse a year before the occurrence of the convulsions, and to this accident the disorder was attributed. The boy was semi-idiotic, with complete paralysis of the lower extremities.

Beardsley observed dribbling of the urine and partial erection of the penis during one convulsion, which attracted his attention to the genitals. The prepuce was non-retractable, the orifice very small, and behind the corona glandis could be felt a ring of some dense substance. There was slight balanitis and adhesion of the mucous surfaces. Circumcision was performed, the mucous membrane torn back, and a ring of dense sebum removed, as hard as ordinary cheese-crust. Recovery was in all respects satisfactory, the young man becoming finally a perfect model of health and strength.

CASE III. A child, 7 years of age, had convulsions, followed in less than ten months by complete paralysis of the lower extremities, being unable to support the body on the limbs, or to move the latter. As the convulsions occurred at night on micturition, the genitals were examined, the foreskin found adherent, and signs of recent inflammation were distinct. Circumcision, removal of pent-up sebum, and rapid recovery followed.

CASE IV. A 10-year-old child had complete paralysis of both lower extremities, convulsions, loss of vision, emaciation and inability to lift the head from the pillow or support the body; he gradually had become idiotic. For three years epileptiform convulsions occurred, upon an average once in three hours. Then followed circumcision, with liberation of a semi-solid ring of sebaceous matter, and rapid recovery so far as the ability to sit up and walk about. Vision, however, (one month prior to the date of report), had scarcely improved.

CASE V. A child, 12 months old, had double internal strabismus, continued rolling motion of head, convulsions, and distinct paraplegia, not so well marked, however, as in the preceding cases. The head could not be supported on the trunk. The epileptiform convulsions were contemporaneous with micturition. An adherent foreskin was excised with the effect of rapid restoration of health and entire cessation of convulsions. Child still under treatment (at date of report) and squinting merely.

In considering cases of this character, a natural surprise is awakened, not so much by the induced phenomena, as by the fact that the trifling cause of diseases so grave in their features and portent, should not more frequently escape all recognition. But yet it is at the portals of the human body that its sentries are stationed—not within its inner chambers. And of all these sentinels, that posted at the sally-port of the external genitals, is the most alert and importunate.

It is unnecessary, in this connection, to cite cases where incontinence of urine—diurnal as well as nocturnal enuresis—has originated in preputial irritation—Trousseau,[1] Black, Pitha, Bryant, Packard, Sweigger-Seidel, and in fact almost every author who has contributed to the literature of the subject, have reported instances of the kind. Their history is, in general, the same, beginning with scolding and whipping, drugging for worms, kidney and bladder complaints, yet always the same continual wetting of bed and body clothing, until finally the surgical character of the disease is recognized. Surely in the light of these facts it becomes the duty of every practitioner who discovers a wet napkin upon a male child over one year old, to enquire as to the cause. Dr. J. H. Pooley has recently published an excellent lecture upon this subject, entitled "Points in the Surgery of Childhood,"[2] in

1 Gazette des Hopitaux, No. 9, 1860.

2 A Series of American Clinical Lectures. Vol. II., No. 9.

which he directs special attention to the subject of nocturnal incontinence. In one of his cases, stillicidium occurred from an overcharged bladder, the distended preputial sac depending from the end of the penis in a semi-transparent bag as large as a hen's egg, "presenting a most astonishing sight, and giving rise to severe pain."

It is, however, quite necessary here to insert the caution, not to conclude that because preputial difficulty may induce nocturnal enuresis, that therefore all nocturnal enuresis is of preputial origin. This is far from being the case, for it is hardly necessary to state that nocturnal enuresis of young male children may be caused by reflex irritation from several organs. I am positively of the opinion that circumcision will completely relieve whenever the prepuce is irritated or irritating, and never otherwise. Only last week I was consulted with reference to a boy six years old, who had been circumcised four years ago, and had regularly wetted his bed every night since. But here the mere inspection of the penis indicated the fact that there had been extra-preputial irritation.

Dr. Brown-Séquard recently made to Dr. F. N. Otis[1] the following interesting statement:

CASE VI. While in London during the past year a gentleman was brought to me who presented all the rational signs of advanced cerebral *ramollissement.* I had looked upon the case as quite a hopeless one, until noticing that the patient frequently applied his hand in an absent sort of way to his genital apparatus. Permission being accorded, examination of the parts revealed an aggravated inflammatory phimosis, complicated with acute balanitis. On making this discovery, I expressed my belief of the possibility that the *apparent ramollissement* might be due to reflex irritation. Complete ablation of the prepuce, with treatment of the balanitis was advised, and practised; and in six weeks thereafter he presented himself at my office, well in every respect.

Dr. Otis' paper presents in the most lucid and forcible manner the results of reflex irritation throughout the genito-urinary tract, induced by contraction of the urethra at or near the urinary meatus, both congenital and acquired. Though the cases reported do not fall within the scope of this article, still they go far to confirm the belief which I desire to insist

1 Ohio Med. and Surg. Jour.. Vol. II., I., p. 16.

upon in connection with the subject—a belief which he expresses as follows: the full significance of this locality as a source of reflex irritation, has not yet been appreciated. A curious illustration of this irritability, was displayed in his 12th case, where constant rhythmical contraction of the cremaster muscles had induced a spasmodic action of the testes, which were carried up and down alternately in the scrotum, with a see-saw motion. Relief was had by division of strictures.

Vulpian[1] is of the opinion that there is no such thing as reflex functional paralysis, and his views are apparently shared by Dr. Jacobi, of New York, who believes that the doctrine of reflex paralysis will be greatly reduced in influence, when a more careful study is given to the cases whose phenomena it is supposed to explain. Thus nervous symptoms, consecutive to lesions of the genitals, are more often referred to contiguous transmission of a morbid process along the nerves upward to the spinal medulla, than, as formerly, to reflex paralysis—the diagnosis of ascending neuritis and myelitis being substituted for the former. In a recent paper on Masturbation and Hysteria in Young Children,[2] he remarks that, in spite of the vigorous and sagacious efforts of such observers as Drs. L. A. Sayre and F. N. Otis, he hesitates to fully accept their views as to the frequency of neuralgic, spastic and paralytic symptoms depending upon phimosis.

These opinions of an observer as judicious and as careful as Dr. Jacobi, are entitled to their full weight. But in the criticism of a selected case which he cites, his objections are chiefly of a negative character—the argument being, that in order to connect a disease with an efficient cause, it is necessary to show conclusively that all other usual causes were not in operation. This is a sound argument in logic, but one which is manifestly weakened in force when we consider another phase of this question, viz., whether when by the removal of a possibly efficient cause an effect ceases, a fair presumption can be raised that some other cause may have operated to produce that effect.

1 Lecons sur l' Appareil Vaso-Moteur. Lecon 17. Paris, 1875.

2 Am. Jour. of Obstet. and Dis. of Women and Child. Feb., and June, 1876.

The criticism is of a case reported by Dr. J. H. Hunt,[1] entitled: Partial paralysis from reflex action caused by adherent prepuce.

CASE VII. The patient, a boy 6 years of age, had a peculiar staggering walk, in which he seemed unable to properly control the lower extremities, and was therefore subject to frequent falls. He was nervous, and exhibited a twitching of the muscles of the face and extremities. His penis was found erected. He started and screamed in his sleep. In an attempt to protrude the tongue, it rolled about the mouth. Articulation and intellect were below the average; in the neighborhood the boy was considered idiotic.

Now Dr. Jacobi objects that the condition of the heart and spine is not stated, nor are any details given as to the previous existence or non-existence of masturbation, or acute articular rheumatism,—facts which should certainly be narrated in giving a history of a case of protracted chorea minor. But for all this, circumcision was followed by rapid improvement.

Dr. Sayre, to whom reference is made above, reported several such cases to the American Medical Association in 1870, and has since contributed largely to the literature of this subject. An illustrative example is given in his recently published lectures on Orthopedic Surgery, which is worthy of note:

CASE VIII. A delicate boy, 5 years old, unable to stand or walk without assistance, had his knees flexed at an angle of forty-five degrees, and Dr. Sayre was summoned to perform subcutaneous myotomy. Convinced that the deformity was of paralytic rather than spastic origin, the surgeon was proceeding to apply the continuous galvanic current over the upper portion of the thighs, when the nurse warned him not to touch the the boy's "pee-pee," as it was "very sore." The latter was in a state of extreme erection; glans small, pointed and tightly imprisoned; meatus, puffy and reddened; orgasm and slight convulsion invariably followed contact. Cireumcision, with removal of dense sebaceous ring, gave complete relief.

Another such instance has recently been recorded by Dr. E. P. Hurd, of Boston,[1] in such fullness that it cannot be here reproduced in detail:

CASE IX. The lad was 7 years old; and the symptoms those of *Ataxie locomotrice*, incoordination of muscular motion, staggering, headlong

1. N. Y. Med. Record, Oct. 16, 1875.
1. Bost. Med. and Surg. Journal, Jan. 18, 1877, p. 68.

pitching, inability to carry food to the mouth, pupils dilated, double outward and upward squint (paresis of third pair of nerves, Jaccoud) and intellectual hebetude. After one severe epileptiform convulsion, a medical man who saw the case in consultation, was inclined to the diagnosis of scrofulous cerebellar disease. The little patient continued to fail rapidly, when an accidental glance revealed an elongated strangulating prepuce, and minute urinary punctum. An operation was followed by complete relief, the only relic of the disturbance being an occasional stiffness and awkwardness of the gait in running.

I shall not dwell upon the fact that the local irritation resulting from preputial stenosis, is often the first link of the chain which results in confirmed habits of masturbation, because there are few professional men who will not assent to the assertion. I am strongly of the opinion that many young men who are carrying about with them an intolerable wound of the moral consciousness, are more sinned against, than sinning. Sinned against, I repeat, merely because it is the duty of every parent to insure his male child a fair entry into the lists of the struggle where the fittest survive, by providing him, as far as possible, with a healthful body. Neglect or evasion of that duty is surely a sin. It is probably true that numbers of boys are taught pernicious practices in schools, but a very large proportion of such cases result from manipulation of the genitals, the incitement to which lies in an irritable prepuce and undue sensitiveness of the mucous membrane. Dr. Jacobi[1] has done excellent service in calling attention to the extremely early age at which masturbation can be practised. In one of his cases, the orgastic excitement seems to have been produced between the ninth and thirteenth month. Physical examination of these little patients, generally reveals red, œdematous, exquisitely tender glans and prepuce, friction upon which by the clothing, when riding in the street cars or upon a rocking-horse, when climbing, when the thighs are approximated or crossed in the sitting posture, (especially upon the floor), or when efforts are made to extricate the male organ from the thick "first pair" of trousers, suffices for the effect.

In adult males the hyperæsthesia of the membrane may

1. Op. cit.

engender a species of pseudo-impotency. I have observed two cases in which this condition was so marked that intromission in coitus was at once succeeded by ejaculation; and marital unhappiness resulted. Doubtless others have been consulted for similar trouble. I have no question that the advisability of circumcision for such patients should be carefully considered, especially when there is præputial retention of semen, such as Erichsen[1] reports that he has known.

True excessive nocturnal spermatorrhœa, as distinguished from undue prostatic secretion under various excitants, is often occasioned by præputial stenosis. The distinction between the two forms is important. It was Ricord who announced that there is a disease worse than syphilis—syphilophobia. One is tempted to add that the youth who, when straining at stool, finds a little glairy prostatic fluid at the meatus, and is thereafter for years confident that he is wasting away with spermatorrhœa, is more to be pitied than the syphilophobist. Subjoined is a single case of my own observation, which illustrates the truth of the statement made above:

CASE X. A gentleman, 35 years old, living in Wisconsin, wrote to consult me regarding a nocturnal spermatorrhœa which was of such frequent occurrence that he was gradually wasting away under its influence. Sexual desire had been wanting for several years—the discharges occurred often six and eight times in the week, without dream or erection. In answer to the full and careful inquiries of my letter, he responded that retraction was impossible, and had always been so. On the strength of my written opinion that an operation might afford him relief, he visited Chicago in June, 1873, and submitted himself to an examination. I removed a rigidly contracted prepuce whose minute orifice was surrounded by tissue of almost cartilaginous hardness, and in three months thereafter, as he remained free from his trouble, he contracted a matrimonial engagement, which (he has since reported by letter) has proved to be the happiest step of his life.

The connection between preputial deformity and intestinal disorder has been noted, so far as I am aware, by but three authors,[2] and that in brief terms. Perhaps, for this reason, a reference to two cases of personal observation, where such

1. Science and Act of Surgery, Phila., 1869, p. 1140.

2. Van Buren and Erichson. Op. cit. Henry, Am. Jour. of Syph. and Derm., 1870, p. 123.

connection seemed evident, may prove of interest. If a child has "ballooning" of the prepuce during micturition, he usually assists the expulsion of urine by straining, and prolapse of the rectum is liable to ensue.

CASE XI. A 3-year-old child had frequent prolapse of the rectum at stool—the bowel descending two inches at times—the discharges being frequently bloody, always painful and accompanied by a fit of crying and straining, from which he recovered in a faint and exhausted condition. Occasionally, instead of voiding feces which were merely stained with blood, he had evacuations which the mother described as consisting of "almost pure blood." The gait was unsteady, the child frequently falling when walking upon a level plane. Prepuce, redundant and slightly puffy—"ballooning" during micturition with expulsive muscular effort The child frequently cried out that a pin was pricking him, pointing at the same time to the genitals; and on such occasions when his clothing was examined, no cause for complaint could be found. While practicing circumcision the preputial fold of mucous membrane was found reddened and almost double the normal thickness. After the operation the child had rectal prolapse and a bloody discharge once only—two days after the operation. He is now in perfect health.

CASE XII. A lad employed by the American District Telegraph Company, aged 8 years, was brought to me by his father in consequence of "lumps in the groin." He was a fine, healthy, rosy-cheeked boy, with an innocent and honest expression of countenance. He was found to be affected with double inguinal hernia, easily reducible. On close questioning I discovered no cause for the rupture, excepting that he had for some time been straining excesstvely at stool—the inguinal swelling having gradually more and more attracted his attention. While manipulating the hernial tumors in the process of their reduction, I observed that as soon as the scrotum or inguinal regions were touched, the penis at once became rigidly erect, and this the instant the touch was repeated. This phenomenon was also observed by Mr. Smith, the instrument maker, who, at my request, took his measurement with a view to applying a double truss. I questioned the lad carefully with reference to his habits, and he affirmed with candor that he had never improperly handled the parts, since all the boys employed by the Company had been warned against the consequences of such a practice. I then, with considerable difficulty, succeeded in retracting a tight prepuce and in extricating from beneath it a dense ring of crust-like sebum, the patient stating that the foreskin had never before been drawn back, and that he had not known such a removal was possible. His father was instructed with regard to the proper cleansing of his boy's penis, and has since reported the radical cure of the hernia, as well as relief from straining at stool, and from the almost constant priapism.

Reliquet[1] furthermore describes a general hysteriform condition associated with preputial irritation, and Van Buren and Keyes cite a case in which all the symptoms of cystitis were induced by a tight prepuce with a small orifice enclosing a sensitive glans. Ablation of the foreskin relieved the patient, who, six years afterward, had no recurrence of his disorder. Coulson[2] gives the similar history of a boy seven years old. Other cases where symptoms of stone in the bladder were relieved by preputial excision, are on record.

The contraindications for circumcision may be briefly stated as those which would, when judiciously considered, forbid surgical interference, in any case.

The apparatus and procedures devised for the operation, have indeed been numerous. Beginning with the Jewish shield or lyre, and the primitive wooden disk of the Arab, the long list includes the devices of Guérin, Cusco and Panas; Blandin's bistoury, with retractable sheath; Nélaton's[2] triblade forceps; Cruise's[4] double blade dilator; Ricord's guarded needle; and Henry's lead lined director, in which the point of a bistoury is buried, after transfixion from without inward.[5] Henry has also constructed a pair of strong, curved, self-closing clamp forceps, for embracing the prepuce. W. R. Gillette, of New York, has succeeded in relieving the stenosis by the introduction of sponge tents. My friend, Dr. R. W. Taylor, also of New York, has an improved pair of scissors for use in the relief of phimosis, produced by chancroidal ulcers.[6] The blades are strong, three inches in length, the lower flattened transversely, at right angles with, and one-tenth of an inch larger than the upper one, rounded smoothly at the end, and resembling an elevator for necrosed bone. Its obvious

1 Traite des opérations des Voies Urinaires, Paris, 1869.

2 Diseases of the bladder. London, 1857.

3 Gaz. des Hôpit., 31, 1868.

4 Dublin Quarterly, 48, p. 482.

5 Am. Jour. of Syph. and Derm. 1870, p. 124.

6 On some practical points in the Treatment of the Phimosis, produced by Chancroidal Ulcers. N. Y., 1872.

use requires no comment. The procedures of Vidal, Dolbeau, Tripier and Cullerier, are described in most of the text books.

The simple but inelegant dorsal incision has the disadvantage of leaving on either side of the glans, two projecting wings or ears, which may become filled with plastic exudation, and seriously interfere with the usefulness of the organ. In one instance I have seen a nodulated mass as large as a hen's egg, depending beneath the frenum, after an incision made in the tissues when in a highly inflammatory condition. Combined with the circular operation, the dorsal incision meets almost all indications.

Pooley makes the useful suggestion to employ multiple sutures, after excision, with fine black silk—the color enabling the operator to remove them afterward with greater readiness. Henry used the continued suture and failed; but it is yet true that multiplicity of sutures, sufficient to give perfect coaptation, is required to secure the best results.

The so-called "backward slip" of the mucous membrane, by which after ablation of the fore-skin proper, a second phimosis is discovered of the membrane embracing the glans, has led to the general method of operating in two steps, successively dividing first the cutaneous, and later the mucous envelope. This should always be resorted to in the case of children.

In adults, the two layers may be simultaneously divided, when they are not agglutinated, by the method of Benjamin Anger, of the Bicêtre Hospital of Paris—his procedure having been recently made public in the French journals. The intended line of section is first traced in ink, when an assistant seizes the prepuce with two pairs of ordinary forceps, one above and one below the glans, being careful to make no traction with them, beyond what is necessary to advance the line beyond the glans. A double whip-cord ligature is then forcibly tightened about the projecting part in advance of the line in ink, and excision practised. There is no hemorrhage, no sutures are required, and no anæsthesia is produced. The pain is sharp and short. Anger claims that in two days his patients are dismissed.

I have operated by slightly modifying Anger's procedure as follows: A Wheeler & Wilson's sewing machine needle held in a pair of needle forceps, or a perineum needle, such as the gynecologists use, with an eye at the point, is threaded with silk, armed with a bit of wax, smeared with vaseline, and carried through the preputial meatus. The foreskin is pierced from within outwards on each side of the glans, at a point midway between the centre of the dorsum and the frenum. The thread is withdrawn from the eye of the needle, the needle brought out as it entered, and after this operation is completed on both sides, traction is made upon the loops of silk until the line in ink is advanced beyond the glans. Then the whip cord ligature is applied as by Anger, and ablation practiced. This method also is, of course, inapplicable, where adhesions have occurred, since the latter preclude the possibility of drawing forward the prepuce.

The treatment of paraphimosis, which is merely an accidental complication of preputial stenosis, requires the greatest consideration in the adult where there are coexistent chancrous or balanitic lesions. I desire merely, in concluding this sketch, to call attention to the fact that when this accident occurs in children, the operation for the so-called relief of stricture, is quite valueless. For actually, even if the nicking of the tissues behind the corona is done, the paraphimosis is not reduced. The reason is that so much of the preputial tissue is distended by the infiltrated swelling, it becomes impossible to draw this swollen tissue over the glans. Of course, when practicable, compression of the glans should be practiced in order to execute the little manœuvre represented by plates in most of the text-books. When this is ineffectual, the parts are best left covered with a cold water dressing merely, and the paraphimosis allowed to take care of itself. I think the annals of the surgery of childhood will be sought in vain, to discover a single case where strangulation and gangrene of the glans followed. That which actually occurs is relief of the præputial stenosis and the paraphimosis at one and the same time. A transverse ulcer soon forms, to liberate the soft tissues of the encircling ring. The infiltrated foreskin thereafter refuses to come forward till resorption has occurred. This is not speedily accomplished, and the consequence is that in the end—and I speak after observation of many cases—the formerly tender and irritable glans becomes indurated so as to endure the friction of the clothing, the prepuce can be worn back of the corona, and nature accomplishes finally what the surgeon aimed to effect by his more brutal bistoury.

NOTE.—Since writing the preceding lines I have received an interesting monograph by Charles Mauriac, of the Hôpital du Midi, Paris, entitled "Mémoire sur le Paraphimosis," in which this subject is exhaustively treated. It was interesting to me to discover on the sixteenth page of this brochure the following remark, which confirms the statement made above—a statement at variance with the dictum of most authors in surgery—respecting the rarity of strangulation and gangrene following paraphimosis. I translate literally:

"I have read in several works that the constriction exercised by the præputial ring upon the subjacent tissues, may be so severe as to compromise the vitality of the glans and of the anterior portion of the corpora cavernosa. They speak of gangrene of the glans resulting from paraphimosis, as quite a common accident. Well, without denying this possibility, I declare that it must be very rare, and for myself I have never seen an example of it, where there were no chancrous complications. Without doubt, interference with the circulation in the glans may be carried to an excessive degree—the latter becoming turgid, violet colored, and in volume twice or three times larger than before. But by its vascular anastomoses with the corpora cavernosa and the spongy portion of the canal, the dangers resulting to it from compression of the dorsal arteries and veins of the penis, are obviated. I repeat, in uncomplicated paraphimosis, the *prepuce alone mortifies*, the glans, though placed in other conditions than those of its normal nutrition, *does not mortify*." [The italics are mine.]

Elsewhere this same author describes the contrast between strangulation in hernia, where the ring never ulcerates while the constricted gut mortifies, and in paraphimosis where the constricting ring generally ulcerates and the strangulated organ almost never. On the twenty.eighth page of his monograph he adds: "Strangulation of the prepuce behind the corona glandis results, so to speak, in the operation for circumcision, half finished." Mauriac supplements this natural operation by surgical interference in the removal of the plastic tissue remaining in the lower limb of the prepuce. But it is to be remembered that he is writing of paraphimosis in adults, and does not especially consider the accident as occurring in male children.

